# Disparities in adherence to diabetes screening guidelines among males and females in a universal care setting: A population-based study of 1,389,697 adults.

**DOI:** 10.23889/ijpds.v7i3.1938

**Published:** 2022-08-25

**Authors:** Padma Kaul, Luan Mahn Chu, Douglas Dover, Roseanne O. Yeung, Dean T Eurich, Sonia Butalia

**Affiliations:** 1 University of Alberta; 2 Alberta Health Services; 3 University of Calgary

## Objectives

National guidelines recommend that all adults over the age of 40 years undergo screening for diabetes at least once every 3-years. We examined the association between sex and adherence to diabetes screening after accounting for age, urban/rural residence and material deprivation. We also examined the subsequent incidence of prediabetes and diabetes in adherent and non-adherent individuals.

## Approach

Our study is based on a retrospective population-level inception cohort of adults aged 40 - 79 years without pre-existing diabetes or cardiovascular disease on April 1, 2013. Data on hospitalizations, emergency department visits, ambulatory clinic visits, physician billing claims, pharmaceutical claims, centralized laboratory data, vital statistics death registry, and census data at the neighbourhood level were linked at the patient level for years 2013 to 2020. Adherence during a 3-year screening period (2013 – 2016) and prediabetes and diabetes during a 4-year follow-up period were examined. Multivariate logistic regression was used to examine the adjusted association between sex and adherence.

## Results

Among 1,389,697 individuals (49.2% male, 50.8% female) adherence rates were 69.9% in males and 79.8% in females. Sex-differences in adherence were largest in younger individuals (58.0% and 72.6% and in males and females aged 40-44 years, respectively) and consistent across rural/urban residence and material deprivation categories. Females were more adherent (adjusted odds ratio 1.93; 95% confidence interval 1.90 to 1.97) than males. Among adherent males and females, 65.3% and 64.8% had at least one Haemoglobin A1C, respectively during the screening period. During the follow-up period, prediabetes and diabetes rates among males who were adherent were 25.9% and 5.2%, compared to 8.8% and 2.1% among non-adherent males; and 20.2% and 2.9% among adherent and 7.3% and 1.3% among non-adherent females.

## Conclusion

Despite lower rates of adherence to screening, males have higher rates of prediabetes and diabetes compared to females. Our study highlights the need to develop education campaigns and targeted interventions at the local and population level to raise awareness and improve diabetes screening rates in young people, especially males.

